# *Chemistry for Dentists*—A Letter to the Senior Editor

**Published:** 1854-01

**Authors:** 


### ARTICLE V.

*Chemistry for Dentists*—A Letter to the Senior Editor.

MY DEAR DOCTOR:

IT cannot fail to gratify those members of the dental profession who are really desirous to see it placed on a scientific basis, to learn that you have determined to introduce a Chemical Department into your Journal. It is well that the oldest dental periodical should take this step. The others probably will soon follow. At any rate, they ought so to do, for as dentists become better educated, mingle more with physicians, and feel the necessity of scientific knowledge, not only to advance their art, but even to retain the position they have assumed as members of a profession which is really a specialty of medicine, they will demand more and more from their teachers, their colleges, their text-books and their journals.

It is to be expected that objections will be made to your course, that sneers will be abundantly thrown out, that small and feeble ridicule will be showered down upon you, but I trust that you will not allow such little things to move you. You have already fought through many innovations, every one of which has redounded to the advantage of the profession, and I hope that you will insist on this with a pertinacity that will bear down this petty and ignorant opposition.

The cry of *cui bono* has already been raised. Hundreds of dentists are unable to see how they can secure more patients and pocket more dollars by possessing a knowledge of chemistry than they could, if destitute of such information. They look at the mere mechanical parts of dentistry and triumphantly ask how chemistry is applicable to them? They can file a tooth, or pull a tooth, or plug a tooth, without any acquaintance with this beautiful science, and that is enough for them. They want nothing more—they will oppose anything more.

But these people do not see that they are retrograding and carrying dentistry back with them to what it was many years ago. They are reducing it again to a mere handicraft, a series of mechanical manipulations without thought or science, and so undoing all that has been done with so much labor and such zeal, within the last twenty years. The question proposed now to dentists, is: Are you content to rank yourselves with the mere artizans, or will you claim a higher position? Will you enroll yourselves among the liberal professions?

The latter can only be done by making dentistry what its intelligent members claim that it is—a specialty of medicine. The teeth have their physiology, their pathology, their therapeutics, their hygiene, just as any other set of organs have. If, then, we should have aurists and oculists, who are received and recognized as men of science by physicians, why should we not have dentists occupying the same position? But be assured, the medical profession, with its learning, its centuries of progress, its high standing in the world, its extensive scope, its varied acquirements, will not accept any specialists who are not themselves scientific. It regards with scorn any mere mechanical act that claims to affiliate itself with it. The physician would as soon think of meeting his cutler upon terms of equality, as one of these ignorant dentists.

There are dentists, however, who clearly understand their true position. Men of scientific attainments themselves, they appreciate them in others, and know how to avail themselves of those truths which lie, as it were, on the outside of their profession. They see that, in making the claim for dentistry, that it shall be considered a specialty of medicine; they take upon themselves to investigate all the questions of physiology, pathology, &c., that can have any connection with the teeth.

To these men, chemistry is all-important. They wish to learn how the various alterations in digestion may affect the nutrition of the teeth. They must first learn, manifestly, what is the composition of an average healthy tooth; how this varies at different ages, in the two sexes, under different circumstances. They must also understand thoroughly the chemical processes of digestion and nutrition, and must look further into the chemistry of the food. The reciprocal action of the teeth and stomach upon one another, is too manifest to need any illustration. Acid matters rejected from the stomach must act upon the teeth. There is, however, a more important relation which needs to be carefully studied and thoroughly comprehended.

Nutrition, in the early periods of life, may be variously impaired. This is manifest from the existence of the various blood diseases. Some of these cachexies are communicated to the fœtus in utero by the diseased blood of the mother. A tuberculous mother gives birth to tuberculous children. The badly organized blood of the parent circulates through the growing tissues and leaves in them its aplastic deposits, or at least the tendency to form them. This morbid condition must, of course, affect all the tissues. The brain suffers, as we learn from the fact that the children of such mothers are extremely liable to hydrocephalus. The glandular system suffers, as shown by the scrofula which so commonly distresses the infancy of these unfortunates. To name no more, the teeth are affected, as every one knows who has paid any attention to the characteristics of a tuberculous diathesis to be found in these organs. Thus we learn, that the nutrition of the teeth is influenced while the fœtus is still in the womb of its mother. The history of such teeth must differ widely from that of those which are perfectly normal in their formation. How are we to learn wherein they differ? Who is to tell us the peculiarity of their composition? The chemist alone. It is to chemistry that we must apply for such information, and if it is not sufficiently learned to answer us, we must direct its inquiries in the proper channel.

Still further, if the food of the newly born infant does not contain the proper proportions of phosphate and other salts, it is very manifest that the development of bones, teeth and nerves will be impossible. We shall have an excess of animal matter and a deficiency of the earths. The consequences of such an error in diet are too manifest to need any explanation. It is clear, however, that there is but one mode to determine this question, and that is to acquire first a thorough knowledge of the chemistry of food.

The food, however, may be sufficient in quantity and suitable in quality, but there may be some derangement in the digestive organs, which will prevent it from being properly introduced into the system. It may ferment in the stomach and produce various compounds, not only innutritious, but actually injurious to the economy. All the organs must suffer from such an unfortunate taint at the fountain head. Here again we need the chemist to explain to us the nature of these alterations of digestion which thus vitiate the nutrition of the whole organism and entail disorder upon every part.

Supposing, however, that these inquires be dismissed as too deep for ordinary investigation, there still remains the important practical fact, that be the causes what they may, there is a marked difference among the different varieties of teeth. Some are hard and resisting ; others are chalky and friable. Some decay with great rapidity; others resist the ordinary agents of destruction for the whole time of a long life. The most exclusively practical man will not deny that the recognition of these varieties and the determination of the probable duration of any given set of organs is a matter of the utmost importance. The cause of decay must be either in the teeth themselves or in the secretions and other fluids which surround them, or what is still more likely, in both. Thus a tooth liable to decay will be destroyed by the common acids of the mouth, and a mouth unusually acid will more rapidly corrode a sound tooth. If these two elements of destruction be combined, the progress of disease will of course be much more rapid.

Now, how are we to get any information in regard to this matter? Manifestly from the chemist alone. It is he who must analyze for us the teeth and the fluids around them, and point out to us the composition of either which produces the result under consideration.

But even should this knowledge be considered unnecessary, it cannot surely be urged that the study of the cause of caries is a piece of mere scientific trifling. "Remove the cause and the effect ceases," is an axiom as old as science. Prevention or prophylaxis is based entirely upon etiology. If our ideas of the cause of any disease be erroneous, our measures of prevention will be futile. Now it is generally regarded as an established fact, that caries is a chemical change induced in the teeth by the action of corrosive agents which surround them.

This view of caries adopted, the question arises: What are these corrosive agents? Let us know them that we may remove them or protect the teeth from their action. This opens up an examination of all the fluids in the mouth with their changes by decay or disease. Foreign substances, which may operate upon the teeth, must also be so analyzed, and their desstructive power accurately measured. Now it is well known that the various articles of our food, during decomposition, generate a great variety of acids, each one of which is a more or less energetic solvent of phosphate of lime, and consequently a destroyer of the teeth. The chemist alone can measure the activity of these agents, and estimate their destructive power, or even determine whether they possess any such power at all. There is a still further question of much importance to the practical dentist. How are these acids effected by the fluids of the mouth? All these questions, the direct practical importance of which it is impossible for the dullest tooth-scraper to avoid perceiving, can be answered only by the chemist.

If we turn to the department of mechanical dentistry we find that it is half made up of chemistry. What causes the occasional crystalline structure of gold, so that it breaks down under the rolls? This surely is a question of some importance to the practical man, who desires to lose neither time nor material. This crystalline compound is exceedingly annoying and it is very desirable to avoid it. It can only be done by understanding the nature of alloys.

Again, it is well known that California gold, directly worked, is not a little injurious to the rolls. This difficulty also exists to some extent in the coin made from this gold. Why is this and how is it to be avoided? The answer to this question must come from the chemist.

Many other questions connected with alloys require the light which chemistry alone can throw upon them. The metals which render gold brittle, the mode of purifying gold, the separation more especially of tin which is peculiarly troublesome, must all be thoroughly understood before a man has a right to lay claim to the character of a good mechanical dentist, because all of this knowledge is directly connected with the very elements of his art.

Still further, we have a thousand questions connected with the reaction of the earths upon one another, the effect of the metallic oxyds upon each other and upon the earths at the high heat of a baking furnace, the changes in these substances induced by the same heat, the methods of obtaining them in perfect purity, the effects of different impurities in the resulting tints, the modes of detecting adulterations, &c. These points are absolutely necessary to be known by the manufacturer of artificial teeth who would work with any certainty, and whose operations are to approach any thing like scientific accuracy. Experiment may indeed reveal these things, but there is a vast difference in economy of time between experiments instituted at random by a man ignorant of chemistry, and those designed by one having a competent acquaintance with that science.

These are facts which I presume no unprejudiced man who knows any thing about the requirements of dentistry will presume to deny. Those who persevere, therefore, in their sneers at chemistry as connected with dentistry, seem to me to be acting very much like those old farmers who talked so much against book agriculture. They had some weight at first, on account of their reputation as practical men, but very soon it was found that book farmers might also be practical, and that they possessed besides an advantage over and above their practical skill, which enabled them to apply it without loss, viz. a scientific knowledge of the principles upon which their art was based.

What has been the result? The mere practical farmer has been crowded out; he has become obsolete, a sort of monument of old fogyism, while scientific agriculture is rising every day higher and higher. So it will be, I venture to predict, with the so called practical dentists who are setting their faces against the introduction of chemical science. The advancement of the art will leave them hopelessly in the rear.

Persevere, then, I pray you, in the course you have laid out for yourself. Though you may encounter opposition at first, you must ultimately triumph, for your cause is a good one.

S******.

*New York, October 16th*, 1853.

